# The clinical impact of the perioperative epidural anesthesia on surgical outcomes after pancreaticoduodenectomy: A retrospective cohort study^☆^^☆☆^

**DOI:** 10.1016/j.sopen.2022.07.004

**Published:** 2022-07-22

**Authors:** Daniel Negrini, Mayan Ihsan, Karine Freitas, Caroline Pollazzon, Jacqueline Graaf, Jorge Andre, Tatiana Linhares, Virna Brandao, Gustavo Silva, Rossano Fiorelli, Patrick Barone

**Affiliations:** a Department of Anesthesiology, Federal University of the State of Rio de Janeiro, Rio de Janeiro, (RJ), Brazil; b School of Medicine, Serra dos Orgaos University Foundation, Teresopolis, (RJ), Brazil; c Department of Anesthesiology, Medical City Teaching Hospitals, Baghdad, Iraq; d School of Medicine, Federal University of Rio de Janeiro, Rio de Janeiro, (RJ), Brazil; e Department of Intensive Care Medicine, National Cancer Institute, Rio de Janeiro, (RJ), Brazil; f Department of Internal Medicine, Unimed Barra Hospital, Rio de Janeiro, (RJ), Brazil; g Department of General Surgery, Federal University of the State of Rio de Janeiro, Rio de Janeiro, (RJ), Brazil; h Department of Anesthesiology, Federal University of Rio Grande do Sul, Porto Alegre, (RS), Brazil

## Abstract

**Background:**

Pancreaticoduodenectomy is a highly invasive procedure associated with high morbidity. Several preoperative variables are associated with postoperative complications. The role of perioperative factors is uncertain. The use of perioperative epidural analgesia is potentially associated with fewer postoperative surgical complications. We hypothesize that perioperative epidural analgesia might be associated with fewer surgical complications.

**Methods:**

We reviewed data from 288 cases performed at our institution between 2012 and 2019, classifying patients into 2 groups: perioperative use of epidural analgesia and non-perioperative use of epidural analgesia. The decision to use epidural as an adjunct to general anesthesia was based on the judgment of the attending anesthesiologist. Uni- and multivariate analyses were then performed to determine factors associated with postoperative surgical complications, ie, postoperative pancreatic fistula, delayed gastric emptying, among others, after adjusting for confounders.

**Results:**

Baseline and intraoperative factors were similar between the groups, except for sex and postoperative surgical complications. In the univariate analyses, factors associated with fewer postoperative surgical complications were the diameter of the pancreatic duct ≥ 6 mm, hard pancreatic gland parenchyma texture, younger age (< 65 years), and perioperative use of epidural analgesia. In the multivariate analyses, perioperative use of epidural analgesia was significantly associated with fewer postoperative surgical complications (odds ratio = 0.31; 95% confidence interval: 0.13–0.75; P = .009), even after adjusting for significant covariates.

**Conclusion:**

Perioperative use of epidural analgesia might be associated with fewer postoperative surgical complications after pancreaticoduodenectomy even after adjusting for pancreatic gland parenchyma texture, pancreatic duct size, and age.

## Main Points

1. The rationale for considering epidural analgesia as a potentially beneficial variable to postoperative clinical and surgical outcomes is based on its possible beneficial role in inflammation and attenuation of the sympathetic response.
2. Pancreaticoduodenectomy is a highly invasive procedure, which is by itself associated with high postoperative complications and morbidity, both clinical and surgical.
3. It is uncertain whether epidural analgesia could have a beneficial effect in the postoperative period after pancreaticoduodenectomy.
4. Even though we have highlighted all possible bias and limitations in our study, we did find a significant association between the perioperative use of epidural analgesia and reduced postoperative surgical complications, according to the Clavien–Dindo Classification, even after adjusting for other factors associated with postoperative complications in the surgical context. We analyzed data from 288 cases performed at our institution between 2012 and 2019 and found that the perioperative use of epidural analgesia was significantly associated with fewer postoperative surgical complications (odds ratio = 0.31; 95% confidence interval: 0.13–0.75; *P* = .009), even after adjusting for significant covariates in a multivariate logistic regression model.
5. Future studies, with a more robust design, should be conducted to clarify the hypothesis that the perioperative use of epidural analgesia is associated with fewer postoperative surgical complications after pancreaticoduodenectomy.

## INTRODUCTION

Pancreaticoduodenectomy (PD) is associated with many postoperative morbid outcomes. The postoperative complication rate varies from 35% to 58% [1], and perioperative mortality is around 2% in reference centers [2]. PD is, however, the standard of treatment for pancreatic adenocarcinoma and periampullary tumors [3]. Because of its high morbidity, a multidisciplinary approach is mandatory to achieve favorable outcomes [4].

Research on the combined use of epidural analgesia and general anesthesia regarding potential effects in the postoperative period has mainly been focused on maintaining adequate pain control and reducing cardiopulmonary complications [5]. Even though some studies suggest that epidural analgesia leads to statically significant lower mean visual-analog pain scores compared to intravenous postoperative analgesia [6], the topic is still controversial [7]. Additionally, failed attempts to administer epidural analgesia are still a significant problem even for experienced clinicians [8].

Recently, the role of perioperative factors in determining the short- and long-term outcomes after surgery has become the focus of research. The regimen of intraoperative fluid administration, the choice general of anesthesia, and the role of perioperative epidural analgesia are potential factors that could alter the clinical outcome of patients, particularly in the setting of more complex and long duration procedures, such as pancreaticoduodenectomy [9].

Evidence suggests that the use of epidural analgesia during perioperative surgery might be associated with postoperative surgical complications [[10], [11], [12]]. One study showed that the rate of postoperative surgical complications, such as those of gastrointestinal and infectious etiology, after PD was reduced with the use of perioperative epidural analgesia [13]. Alternatively, because perioperative use of epidural analgesia might be associated with hemodynamic instability, there is a possibility that a patient would need a higher dose of vasopressors and higher volume of fluid administration. Arterial hypotension has been reported to be associated with ischemia-related events, such as acute renal failure [14]. Indeed, some evidence has shown a significant association between perioperative use of epidural analgesia and higher postoperative gastrointestinal complications after major abdominal surgeries [15].

Based on the current state of the knowledge, the use of perioperative epidural analgesia and its association with short-term outcomes after PD still need to be clarified. Additionally, the potential role of other covariates in affecting the association between perioperative use of epidural analgesia and postoperative surgical complications after PD is still unclear. Moreover, previous studies did not consider the effects of well-known surgical variables, such as pancreatic gland texture and duct size, in their analysis.

The goal of the present study was to determine the clinical impact of the use of perioperative epidural analgesia in short-term postoperative surgical complications after PD, adjusting for other relevant covariates.

## METHODS

This was a retrospective, observational cohort study of patients who underwent pancreaticoduodenectomy in our institution. We performed chart review of 288 cases performed between January 2012 and December 2019. This study was approved by our Ethical Committee (# 51016121.8.0000.5258). Also, this study adhered to the STROBE checklist for reporting of cohort studies (Appendix Material).

Epidural analgesia is usually achieved by injecting a combination of local anesthetics and opioids into the epidural space. These agents inhibit neurotransmission and nociceptive input. Therefore, it is assumed that epidural analgesia can reduce inflammation and lower the titer of circulating cytokines [16]. Additionally, epidural analgesia can reduce the tone of the sympathetic nervous system, leading to vasodilation [17].

**Fig 1:**
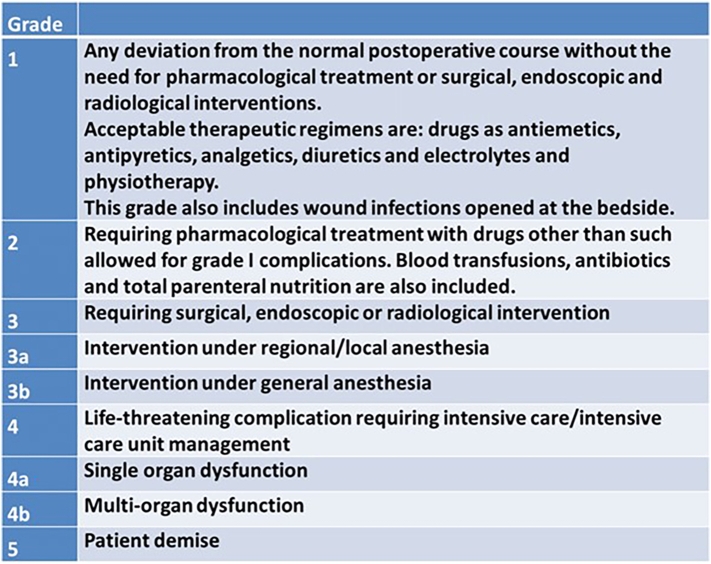
Clavien–Dindo classification for postoperative surgical complications. Grades I and II → minor complications versus grades III or greater → major complications.

The primary outcome measure was the occurrence of any postoperative surgical complication, according to the Clavien–Dindo classification (Fig 1). Postoperative surgical complications most likely associated with PD included postoperative pancreatic fistula (POPF), delayed gastric emptying (DGE), hemorrhage, infection, bile leak, thrombotic events, and hernia, with the first two being by far the most significant. We also collected data regarding the perioperative use of epidural analgesia and other factors we reasonably assumed could impact the relationship between the perioperative use of epidural analgesia and our outcome of interest, such as the total number of intraoperative fluids used, type of fluid used (crystalloids only or both crystalloids and colloids), and intraoperative use of vasopressors. Data regarding the texture of the pancreatic gland parenchyma and pancreatic duct size were also collected because the relationship between pancreatic gland texture/duct size and postoperative surgical complications has been well established. A soft pancreatic texture and ductal size of ≤ 3 mm are associated with higher risk of postoperative complications such as POPF. The soft pancreas and small ductal size are significantly relevant factors in the Fistula Risk Score based on the 2005 and 2016 International Study Group of Pancreatic Fistula classification [18]. Patients with serious cardiovascular or pulmonary diseases were excluded from the study. We also collected data regarding demographic characteristics and preoperative diagnosis.

From 2012 to 2019, 288 patients underwent PD at our institution and were included in this study. Subjects were then classified into epidural and nonepidural groups. The decision to use epidural as an adjunct to general analgesia or not was based on the judgment of the attending anesthesiologist. In our institution, high complex surgeries such as pancreaticoduodenectomy have analgesia care performed by few clinicians, which could limit the bias related to the decision of using or not intraoperative epidural analgesia. In our institution, epidural analgesia for PD cases is usually performed with a Tuohy-type needle, with a median approach, at low thoracic level. Also, ropivacaine 0.2% is used as the local anesthetic of choice, administered intermittently, alongside with 2 mg of morphine. The dose of ropivacaine is dictated individually during the intraoperative period. In the postoperative period, local anesthetic and opioid epidural administration is guided by individual patient needs, as judged by the clinician evaluating the patient the day after surgery. Usually, epidural catheters are removed the day after surgery. Also, most cases of PD in our institution are performed under total intravenous anesthesia, with similar drugs both in the intra- and postoperative period. Postoperative analgesia is also complemented with dipyrone and tramadol, unless contraindicated.

### Statistical Analysis

Univariate and multivariate analyses were conducted to assess factors significantly associated with our outcome of interest. For continuous variables, we used unpaired 2-sample *t* test (2-tailed) for group comparisons. For categorical variables, we used the *χ^2^* test. For the uni-and multivariate analyses, we used logistic regression. For the multivariate analysis, we used forward selection of variables, starting with the one with the lowest *P* value on univariate analysis, ending only with the variables with *P* values less than .05 in the univariate analysis. We had previously estimated a sample size of 140 patients (70 in each group) to power the study to detect a difference in any grade postoperative surgical complications of at least 20% between groups (80% power), assuming a type I error (α) of .05, accounting for 5 predictors in a multiple regression model. We used the free online software G-Power for sample size calculation. The final inclusion number of 288 cases was higher than the estimated sample size needed to power the study to detect significant biostatical differences between groups. All analyses were performed using Stata version 15.1 (StataCorp LLC, College Station, TX, USA)

## RESULTS

Baseline demographic and preoperative factors did not have statistical difference between the epidural and nonepidural groups, except for sex (Table 1). Their intraoperative and postoperative factors also did not statistically differ between both groups, except for surgical complications (*P* = .005) (Table 2). Nearly half of the complications in the epidural group consisted of DGE and postoperative pancreatic fistula. In the nonepidural group, those 2 complications nearly comprised two-thirds of the total complications. Other postoperative complications, such as bleeding and intra-abdominal collection, were individually in small numbers. Thus, we decided to group then as "others." Additionally, estimated blood loss and the use of blood products did not differ between groups (Table 2). The uni- and multivariate analyses of factors potentially associated with the occurrence of any postoperative surgical complication are shown in Table 3. In the univariate analyses, perioperative use of epidural, hard pancreatic gland texture, and pancreatic duct diameter ≤ 6 mm and younger age (≤ 65 years) were all associated with fewer postoperative surgical complications. In the multivariate analyses, perioperative use of epidural analgesia was still significantly associated with fewer postoperative surgical complications (odds ratio [OR] = 0.31; 95% confidence interval [CI]: 0.13–0.13; *P* = .009), along with hard pancreas (OR = 0.39; 95% CI: 0.21–0.72; *P* = .003) and age ≤ 65 years (OR = 0.54; 95% CI: 0.31–0.93; *P* = .026).

**Table 1:** Demographic, preop diagnosis, and pancreas characteristics of epidural and no-epidural groups

	*Epidural (*n *= 240)*		*No epidural (*n *= 48)*		*Total (*N *= 288)*		
	n *or mean*	*% or SD*	n *or mean*	*% or SD*	n	*%*	P
Sex							
Female	103	42.92	29	60.42	132	45.83	.026
Male	137	57.08	19	39.58	156	54.17	
Age	65.79	11.85	65.37	11.66			.818
BMI	26.22	5.74	26.18	6.10			.972
Pancreatic gland texture							
Hard	106	44.17	23	47.92	129	44.79	.633
Soft	134	55.83	25	52.08	159	55.21	
Pancreatic duct size							
< 3 mm	35	14.58	9	18.75	44	15.28	.721
3–6 mm	127	52.92	23	47.92	150	52.08	
> 6 mm	78	32.5	16	33.33	94	32.64	
Diagnosis							
Cancer (vs benign)	38	79.17	171	71.25	209	72.57	.436
NET (vs benign)	4	8.33	20	8.33	24	8.33	
Benign	6	12.5	49	20.42	55	19.1	

*BMI*, body max index; *NET*, neuroendrone tumor.

**Table 2:** Intra- and postoperative characteristics of epidural and no-epidural groups

	*Epidural (*n *= 240)*		*No epidural (*n *= 48)*		*Total (*N *= 288)*		
	n *or mean*	*% or SD*	n *or mean*	*% or SD*	n	*%*	P
Any surgical complications							
Yes	155	64.16	41	85.11	190	67.77	.005
No	85	35.84	7	14.89	94	32.23	
Type of surgical complication							
DGE	59	38.31	17	35.42	58		.153
Fistula	48	31.16	18	43.90	39		
Other	47	30.53	13	20.68	50		
Amount of fluids (ml/kg.h)	7.93	2.66	8.22	3.33			.557
Type of fluids							
Crystalloids	81	34.05	19	40.54	100	35.14	.451
Both crystalloids and colloids	159	65.95	27	59.46	188	64.86	
Vasopressors were used intraop.							
Yes	229	95.68	48	100.00	277	96.4	.198
No	11	4.32	0	0.00	11	3.6	
Estimated blood loss	494.65	582.21	425.00	288.79			.4196
Length of hospital stay	12.87	8.16	12.80	7.30			.9596
Length of ICU stay	1.60	1.83	1.79	1.75			.5022
Readmission to ICU in 90 d							
Yes	88	25.99	15	31.82	103	28.49	.436
No	152	74.01	33	68.18	185	71.51

**Table 3:** Uni- and multivariate analysis of factors affecting surgical complications

	*Univariate analysis*					*Multivariate analysis*				
	*OR*	*95% CI*			*P*	*OR*	*95% CI*			*P*
Male sex	0.92	0.55	–	1.53	.744					
Age > 65 y	1.8	1.08	–	3.005	.025	1.856	1.077	–	3.201	.026
BMI > 25	1.21	0.73	–	2.01	.468					
Amount of fluids > 8 ml/kg.h	0.79	0.47	–	1.33	.375					
Type of fluids was both crystalloids and colloids	0.80	0.59	–	1.09	.158					
Vasopressors were used intraop.	4.38	0.78	–	24.55	.093					
Estimated blood loss was > 400 ml	0.97	0.58	–	1.61	.900					
Perioperative use of epidural	0.31	0.13	–	0.73	.007	0.31	0.13	–	0.75	.009
Pancreatic gland texture was soft	2.75	1.62	–	4.64	<.001	2.54	1.38	–	4.67	.003
Pancreatic duct size			–							
< 3 mm (vs > 6 mm)	0.42	0.19	–	0.91	.029	0.64	0.25	–	1.60	.340
3–6 mm (vs > 6 mm)	0.52	0.28	–	0.97	.040	0.78	0.39	–	1.56	.479
> 6 mm	1.00		–							
Diagnosis			–							
Cancer (vs benign)	0.79	0.41	–	1.55	.496					
NET (vs benign)	1.22	0.38	–	3.94	.856					
Benign	1.00		–							
Duration of surgery > 400 min	0.90	0.53	–	1.53	.703					

## DISCUSSION

Recently, focus of research been placed on potentially modifiable perioperative factors that might impact immediate short- and long-term postoperative surgical outcomes. The adoption of protocols like ERAS by many institutions worldwide shows the extent of attention this topic has acquired in the past years [19]. The use of perioperative epidural analgesia is important in the implementation of those protocols, as it assumingly leads to better postoperative pain control and, consequently, faster return to baseline physiologic functions, such as ambulation and ventilation. Postoperative complications, such as thromboembolic events, atelectasis, and pulmonary infections, and major adverse cardiac events may be reduced because of these beneficial physiological effects [20]. However, results from previous studies are, at best, controversial. [21].

Additionally, in the scenario of colorectal surgery, the use of perioperative epidural analgesia along with opioids has been associated with some drawbacks, such as nausea, delayed return to normal bowel function, and longer length of hospital stays [22]. Moreover, hemodynamic instability is sometimes associated with epidural analgesia, which could result in an increased need for fluid and vasopressor administration and worse overall postoperative outcomes after major pancreatic surgeries [23]. In our study, we found no significant difference in the use of vasopressor, blood loss, or total amount of fluid used between groups.

Some evidence tends to favor the hypothesis that epidural analgesia blunts the surgery-related inflammatory cascade, leading to lower levels of circulating cytokines [24]. It is unclear if the proposed mechanism might affect the occurrence of postoperative surgical complications. Evidence from animal models have shown an association between reduced inflammatory response and the use of epidural analgesia, and fewer occurrence of metastasis in cancer patients, consequently to the modulatory effect on the inflammatory cascade [25]. In our study, we merely showed an association between the use of intraoperative epidural analgesia and short-term minor (Clavien–Dindo grades I and II) postoperative surgical complications. The mechanisms behind this phenomenon need to be explained by future randomized controlled trials.

In our study, we failed to find any significant effects from the use of intraoperative epidural analgesia on some clinically relevant postoperative outcomes, such as length of hospital stay, length of intensive care unit (ICU) stay, and readmission to the ICU (Table 2). However, we succeeded in proving an association between intraoperative use of epidural analgesia and fewer short-term postoperative complications.

PD is a highly invasive and long duration procedure, associated with many postoperative complications, including POPF. Some preoperative factors are known to be associated with postoperative surgical complications. Studies have described the texture of the pancreas as an independent predictive factor for the occurrence of POPF and other pancreatic surgery complications. Pancreatic fibrosis and POPF occur more frequently in soft-textured pancreases [26] and are associated with increased pancreatic fat [27]. Our results are consistent with the literature, with soft pancreas being significantly associated with postoperative surgical complications. Conversely, hard-textured pancreas that develop from fibrosis are associated with lower POPF formation, as these pancreases allow firmer holding of sutures and tend to have a smaller amount of pancreatic juice secretion. Pancreatic texture is typically assessed by a surgeon during surgery, but several experimental approaches have yet to gain approval for clinical application [27]. Additionally, the diameter of the main pancreatic duct is known to be associated with postoperative surgical complications [28]. One could argue that "soft pancreas" itself is already a strong predictor of postoperative surgical complications, so the use of intraoperative epidural analgesia would not matter. Our data indeed show, in the univariate analysis, that "soft pancreas" is associated with more postoperative surgical complications. However, in the multiple logistic regression model, even adjusting for the covariate "soft pancreas," intraoperative use of epidural analgesia was still associated with fewer postoperative surgical complications.

Some studies have also shown that age of ≥ 65 years, as well, is associated with higher morbidity and mortality after PD [29]. Our group has not found this association between older age and postoperative surgical complications. This study reveals that all covariates, such as age, parenchymal texture, and size of the pancreatic ducts, did not differ between epidural and nonepidural groups, and intraoperative epidural use was associated with minor surgical complications after adjusting for all those factors.

The results of the current study are consistent with the hypothesis that perioperative use of epidural analgesia might be associated with fewer postoperative surgical complications, at least Clavien–Dindo grades I and II (minor). It is also interesting to note that the rate of postoperative surgical complications is not associated with the average total volume of fluids administered, the type of fluid administered, or the use of vasopressors, often considered potential confounders in the association between perioperative use of epidural analgesia and surgical outcomes [30]. Those factors were similar between epidural and nonepidural groups in our study.

Difficulty in establishing a causal relationship and mechanism between the perioperative use of epidural analgesia and postoperative surgical complications is a potential limitation of our study. Additionally, one could argue that the decision to use epidural analgesia, or any other instrument to improve the quality of anesthetic care by the attending anesthesiologist, would imply a different perception of the impact of more accurate intraoperative anesthetic care in postoperative outcomes and potentially lead to less postoperative complications. This should also be considered an additional potential source of bias, although the use of intraoperative epidural analgesia is the standard of care in our institution for PD cases, unless contraindicated, and anesthesia care for complex cases, such as PD, are performed by few professionals. After considering all the limitations of our study, we certainly must be extremely careful in any analysis and interpretation of the present findings and the extent of how far we can extrapolate our conclusions. Consequentially, based on the strength and significance of the evidence we found, future randomized clinical trials on this topic should be performed, especially considering the role of other potential covariates known to be associated with postoperative surgical complications after pancreaticoduodenectomy.

Based on the data from our institution, we conclude that the use of perioperative epidural analgesia might be associated with fewer minor postoperative surgical complications after pancreaticoduodenectomy, ie, Clavien–Dindo grades I and II, even after adjusting for factors known to be associated with postoperative surgical complications. Facing a yearly increase in the number of complex surgical procedures, anesthesiologists and surgeons need to be aware of the importance of intraoperative care and its significant relevance to surgical outcomes.

The following are the supplementary data related to this article.

Appendix Fig 2. STROBE Statement—checklist of items that should be included in reports of *cohort studies.* Appendix

## Author Contribution

DN and MI: conception and design and writing the article.

KF, CP, and JG: data collection.

JA, TL, and GS: analysis and interpretation.

VB and PB: critical revision.

## Conflict of Interest

The primary author and all co-authors of the present study declare that they have no conflict of interest in this investigation. We hereby accept the terms of the conflict on interest form.

## Funding Source

The primary author and all co-authors of the present study declare that there was no funding source during this present investigation.

## Ethical Approval Statement

The primary author and all co-authors of the present study declare that they have complied with all ethical aspects concerning the confidentiality of the patient information and that they intend to keep all data unidentified. The present investigation was also approved by our local ethical committee, as stated in the methods section of the manuscript.
